# The effect of nasal septum surgery on anterior maxillary teeth sensation – a prospective study

**DOI:** 10.1007/s00405-025-09469-8

**Published:** 2025-06-09

**Authors:** Dan Littner, Shibli Alsleibi, Elena Pichkhadze, Nir Abraham Gecel, Hadas Knoller, Shay Izhak Duvdevani

**Affiliations:** 1 Department of Endodontics, The Maurice and Gabriela Goldschleger School of Dental Medicine, Gray Faculty of Medical & Health Sciences, Tel Aviv University, Tel Aviv, Israel; 2 Department of Otolaryngology - Head and Neck Surgery, Sheba Medical Center, Derech Sheba 1, 52621 Ramat-Gan, Israel; 3 Gray School of Medicine, Gray Faculty of Medical & Health Sciences, Tel Aviv University, Tel Aviv, Israel

**Keywords:** Nasal septum, Nasal septum surgery, Teeth sensation, Palate sensation, Anterior maxillary sensation

## Abstract

**Introduction:**

Nasal septum surgery has been associated with postoperative numbness or paresthesia of the anterior maxillary teeth, primarily involving the incisors and canines, with an incidence reported between 3% and 30%. This sensory alteration may result from trauma to the nasopalatine nerve or anterior superior alveolar nerve, which innervate the anterior maxilla. Current literature on this topic is limited, outdated, and primarily based on retrospective studies that lack long term follow-up. This study aims to investigate the impact of nasal septal surgery on anterior maxillary teeth sensation, focusing on the incidence, predisposing factors, and the timeline for sensory recovery.

**Methods:**

This prospective study included 21 patients who underwent nasal septum surgery from September 2023 to September 2024. Patients with pre-existing conditions affecting dental sensation or those who recently underwent dental procedures were excluded. Sensory assessments were conducted preoperatively, post-topical anesthesia application, and at 10 days, one month, and three months post-surgery. Demographic data, surgical approach, and medical history were recorded.

**Results:**

The cohort had an average age of 32.6 years (range 20-62), with 52% male participants. Significant temporary sensory loss was observed following the application of topical anesthesia (p=0.007). Postoperatively, sensory reduction occurred in two patients, resulting in a 9.5% incidence of sensory disturbances (p=0.4): one patient experienced numbness in all four incisors, while another reported numbness in the two central incisors. Both patients regained full sensation within one month after surgery. Multivariate analysis did not identify any significant predictive factors for sensory impairment, including surgical approach, presence of nasal fracture, or history of prior nasal surgeries.

**Conclusion:**

Nasal septal surgery is associated with a low incidence of transient sensory disturbances in the anterior maxillary teeth, with full recovery typically occurring within one to three months. These findings suggest that careful surgical technique can minimize nerve injury and that the risk of permanent sensory loss is low. Additional research with larger patient cohorts is needed to validate these findings and identify possible risk factors, thus improving patient counseling and surgical techniques.

## Introduction

Nasal septum surgery can lead to postoperative numbness or paresthesia affecting the anterior maxillary teeth, particularly the incisors and canines, with a reported incidence ranging from 3 to 30% [1, 2]. This may arises from injury to the arterial supply or nerve branches innervating the anterior maxilla [3]. The primary nerves involved in the innervation of the anterior maxillary teeth are the anterior superior alveolar nerve and the nasopalatine nerve.

The anterior superior alveolar nerve branches off from the infraorbital nerve in the anterior portion of the infraorbital canal. It descends along the anterior wall of the maxillary sinus, where it innervates the mucosal lining. From there, the nerve penetrates the alveolar process of the maxilla, forming a dental plexus that supplies the anterior maxillary teeth. It also gives rise to small nasal branches that innervate the inferior nasal cavity and communicates with fibers from the middle superior alveolar nerve within the maxillary dental plexus [4].

The nasopalatine nerve originates from the maxillary nerve in the pterygopalatine fossa and passes through the sphenopalatine foramen to enter the nasal cavity [5]. It travels below the sphenoid sinus ostium to reach the nasal septum and descends anteroinferiorly between the periosteum and the mucous membrane of the nasal septum, running in a groove on the vomer bone. After merging with its counterpart on the opposite side, the nasopalatine nerve exits the nasal cavity through the incisive canal and emerges onto the palate through the incisive foramen. Here, it communicates with the greater palatine nerves [6, 7]. The nasopalatine nerve provides sensory innervation to the mucosal surfaces of the palate and nasal septum, as well as to the six anterior maxillary teeth [8].

Sensory impairment of the anterior palate is often linked to the use of a chisel on the maxillary crest, which can potentially damage the nasopalatine nerve as it enters the incisive foramen. Moreover, teeth paresthesia may occur due to intraoperative septal dislocations close to the anterior nasal spine, nasal septum cartilage resections or anterior nasal spine remodeling procedures [9]. This numbness is typically transient and resolves within weeks to months [2]. However, the use of cautery near the nasopalatine foramen may result in more prolonged sensory deficits [1].

The medical literature on dental complications following septum surgery is limited, outdated, and primarily based on retrospective studies [1, 10]. In this study, we aim to prospectively investigate the occurrence of teeth numbness after nasal septum surgery, identify potential predisposing factors, and determine the timeline for the return of normal sensation.

## Methods

The primary objective of this study was to evaluate the incidence of postoperative numbness in the six anterior maxillary teeth following nasal septal surgery and to determine the timeline for sensory recovery. Between September 2023 and September 2024, we enrolled 21 patients who underwent nasal septal surgery at a tertiary medical center. Eligible participants were adults aged 18 years or older who had undergone nasal septal manipulation. We excluded patients under the age of 18, those who underwent nasal surgery without septal manipulation, patients with dental implants, edentulous patients, individuals with recent dental procedures, patients with caries, restoration, periapical lesions or resorptions, and those with known oral or dental diseases that could affect sensation.

This study was conducted with approval from the Helsinki Ethics Committee, and all participants provided informed consent. Demographic information, including gender, age, general medical history, and the reasons for undergoing surgery, was obtained from the medical center's database. We also recorded whether patients had a history of nasal fractures or previous nasal surgeries. Specific surgical details were collected, including the type of surgical approach (Killian incision, hemitransfixion, or open rhinoplasty) and whether osteotomies or turbinectomies were performed.

Each patient underwent a preoperative dental assessment by a endodontics specialist. The evaluation included mobility, palpation, percussion, and assessment for the presence of caries, sinus tracts, periodontal disease, color changes, periapical lesions, or previous root canal therapy. We used the cold pulp test to assess sensation in the six anterior maxillary teeth (two canines and four incisors). In fact, previous research has demonstrated that, among various sensibility tests, the electrical pulp test offers the most reliable accuracy. It is important to note that a negative response does not necessarily indicate pulp necrosis, since sensibility tests measure nerve activity rather than the vascular supply, which is the key factor in pulp survival [11, 12].

Patients underwent preoperative assessment 7–10 days before surgery. Following this initial evaluation, topical anesthesia (lidocaine or amethocaine with adrenaline) was applied to the nasal area for 30 min, followed by a reassessment of tooth sensation. Postoperatively, tooth sensation was evaluated at 10 days, one month, and three months following surgery. Patients with normal postoperative sensation were not tested further, while those with diminished sensation continued to be evaluated until sensation returned to their baseline state. This allowed us to document the time course of sensory recovery following surgery.

## Results

A total of 21 participants were included in the study, with 11 males (52%) and an average age of 32.6 years (range 20–62 years). Of the participants, 57% were medically free, while 14% had asthma. These demographics align with the typical profile of patients seeking septal surgery to improve nasal breathing, as most are young and generally healthy.

The most common presenting symptom was difficulty with nasal breathing (81%), followed by nasal discharge (24%), olfactory dysfunction (19%), facial pain (19%), and atopy (19%). Additionally, 24% of patients had a history of nasal fracture, and 19% had undergone previous nasal surgery.

In terms of surgical approach, 55% of patients underwent surgery using a hemitransfixion or full transfixion incision, while 45% had a Killian incision. No patients in this cohort underwent surgery with an open rhinoplasty incision. Turbinate surgery was performed in 60% of cases, and osteotomies were done in 45% of patients.

Table 1 provides detailed information regarding the demographic and patients characteristics.

**Table 1:** Patients’ Characteristics and Demographics, Symptoms, and Intraoperative approach

Variable	Sample
Gender:	
Males, *n* (%)	11 (52%)
Females, *n* (%)	10 (48%)
Age (years), (range)	32.6 (20–62)
Symptoms:	
Difficult nasal breathing	81%
Nasal discharge	24%
Olfactory dysfunction	19%
Facial pain	19%
Atopy	19%
Previous nasal fracture	24%
Previous nasal surgeries	19%
Incision	
Hemitransfixion, full transfixion incision	55%
Killian incision	45%
Open Rhinoplasty incision	0%
Turbinate surgery	60%
Osteotomies	45%

Data are expressed as numbers and percentage

The data in Table 2 indicates a significant reduction in teeth sensation following the application of topical nasal anesthesia (*p* = 0.007). However, no significant reduction in sensation was observed postoperatively (*p* = 0.4).

**Table 2:**
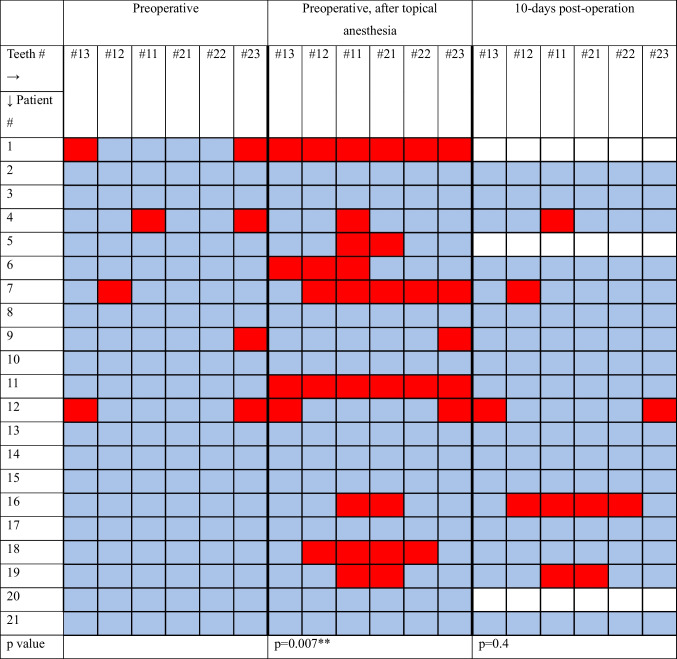
Teeth Sensation at Three Timelines—preoperative (before the operation), preoperative after the application of topical nasal anesthesia, and 10 days postoperation. Each tooth is listed in a specific column and numbered according to the FDI World Dental Federation (ISO) notation (see below *). Each patient is represented in a separate row, with their teeth sensation recorded across the three timelines using color-coded squares: blue indicates normal sensation, red indicates reduced sensation, and white indicates not tested

(*) Teeth numbers and Names:

- Tooth #13: Upper right first premolar

- Tooth #12: Upper right lateral incisor

- Tooth #11: Upper right central incisor

- Tooth #21: Upper left central incisor

- Tooth #22: Upper left lateral incisor

- Tooth #23: Upper left first premolar

Remarkably, all patients who experienced sensory changes postoperatively also had teeth numbness after the application of topical anesthesia. Furthermore, it is important to note that patients #4, #7, and #12 had preexisting reduced sensation in four specific teeth before surgery (as highlighted in yellow in Table 3) and continued to exhibit reduced sensation in these same teeth during follow-up assessments. Consequently, this leaves only two patients, #16 and #19, who demonstrated actual postoperative sensory reduction. At postoperative day 10, patient #16 had numbness in all four incisors, while patient #19 had reduced sensation in the two central maxillary incisors. One month after surgery, Patient #16 had regained sensation in one of the affected incisors, while Patient #19 had fully recovered normal sensation. At three months patient #16 also had full recovery in teeth sensation (Table 4).

**Table 3:**
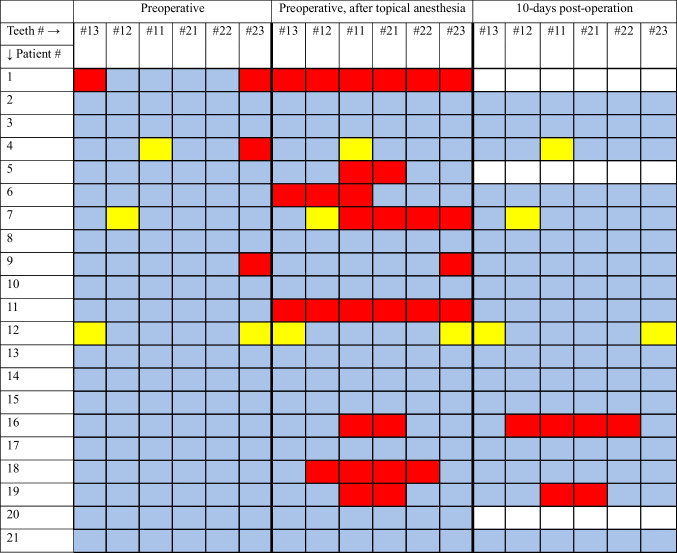
Teeth Sensation at Three Timelines—preoperative (before the operation), preoperative after the application of topical nasal anesthesia, and 10 days postoperation. Each tooth is listed in a specific column and numbered according to the FDI World Dental Federation (ISO) notation (see below *). Each patient is represented in a separate row, with their teeth sensation recorded across the three timelines using color-coded squares: blue indicates normal sensation, red indicates reduced sensation, and white indicates not tested. Yellow squares highlight patients who exhibited reduced sensation in specific teeth prior to surgery and continued to show diminished sensation in these teeth during subsequent tests. Excluding patients with yellow squares leaves only two patients (Patient #16 and Patient #19) who demonstrated actual postoperative sensory reduction

(*) Teeth numbers and Names:

- Tooth #13: Upper right first premolar

- Tooth #12: Upper right lateral incisor

- Tooth #11: Upper right central incisor

- Tooth #21: Upper left central incisor

- Tooth #22: Upper left lateral incisor

- Tooth #23: Upper left first premolar

**Table 4:**
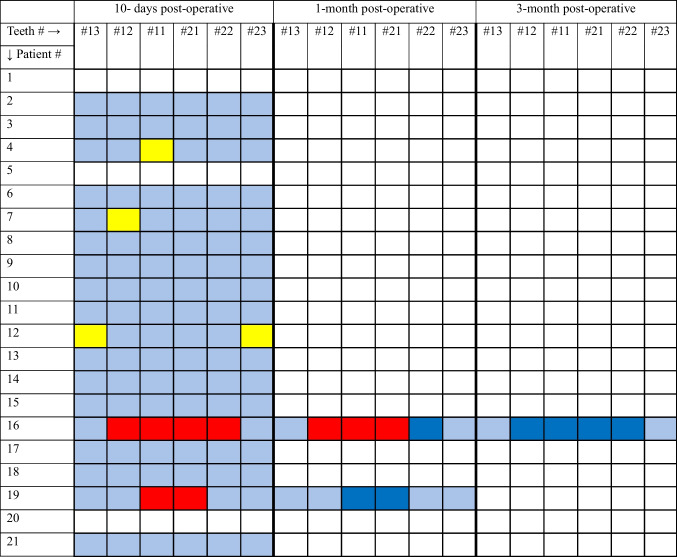
Teeth Sensation at Three Timelines—10 days postoperation, 1 month postoperation, and 3 months postoperation. Each tooth is listed in a specific column and numbered according to the FDI World Dental Federation (ISO) notation (see below *). Each patient is represented in a separate row, with their teeth sensation recorded across the three timelines using color-coded squares: blue indicates normal sensation, red indicates reduced sensation, white indicates not tested, and Yellow squares indicates patients who exhibited reduced sensation in specific teeth prior to surgery and continued to show diminished sensation in these teeth during subsequent tests. Dark blue highlights patients who experienced reduced sensation on postoperative day 10 but recovered sensation in subsequent tests at 1 month or 3 months postoperation

(*) Teeth numbers and Names:

- Tooth #13: Upper right first premolar

- Tooth #12: Upper right lateral incisor

- Tooth #11: Upper right central incisor

- Tooth #21: Upper left central incisor

- Tooth #22: Upper left lateral incisor

- Tooth #23: Upper left first premolar

**Fig. 1:**
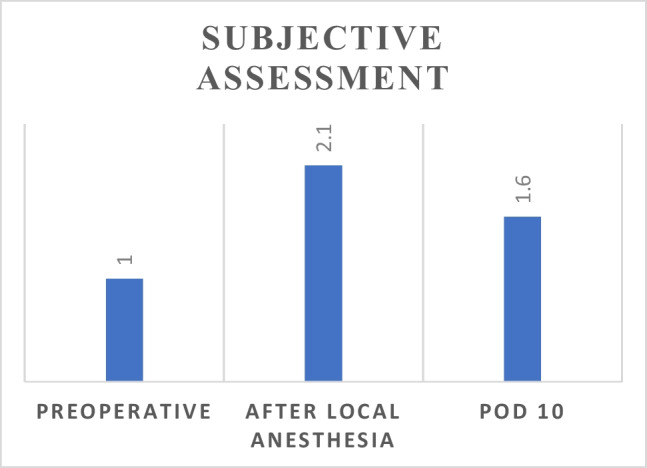
Patients’ subjective perception of their teeth sensation recorded at three time points: preoperatively, after the application of topical anesthesia, and on postoperative day 10. Sensation was rated on a scale of 1 to 5, where 1 represents normal sensation and 5 represents a complete loss of sensation

**Fig. 2:**
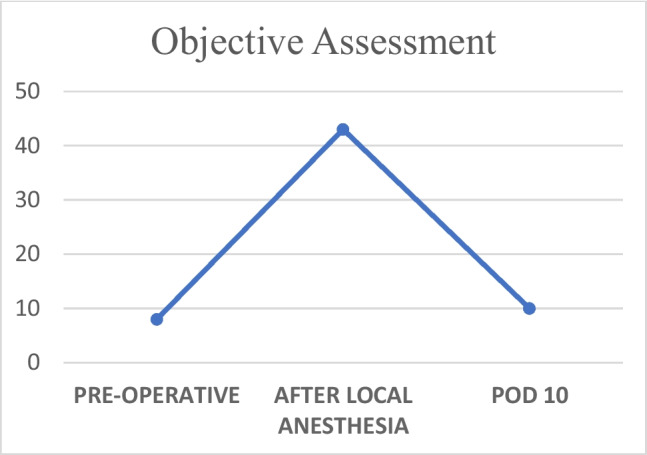
Objective assessment of teeth sensation recorded at three time points: preoperatively, after the application of topical anesthesia, and on postoperative day 10. The y-axis represents the number of teeth with diminished sensation

Patients were asked to provide a subjective assessment of their teeth sensation at three time points: preoperatively, after the application of topical anesthesia, and on postoperative day 10. This was done using a scale of 1 to 5, where 1 indicated normal sensation and 5 indicated complete loss of sensation. As shown in Figs. 1 and 2, the subjective assessments were consistent with the objective findings from our sensory testing, demonstrating a clear correlation between the patients'perceptions and the actual measured changes in teeth sensation.

We conducted a multivariate analysis to determine whether any factors could predict which patients might experience postoperative sensory changes following septal surgery. However, no factor was found to be statistically significant (Table 5).

**Table 5:** Multivariate Analysis of Factors Predicting Postoperative Diminished Teeth Sensation. This analysis examines whether these factors can predict reduced sensation following surgery

Variable	*p*-value
History of nasal fracture	0.452
Previous nasal surgery	1.8
Incision type	0.542
Anterior septal deviation	0.68
Bony deviation	0.67
Turbinate surgery	0.98
Osteotomy	0.52

## Discussion

This study aimed to evaluate the incidence and course of sensory changes in the anterior maxillary teeth following nasal septum surgery. Our findings indicate that the incidence of postoperative sensory disturbances was low. Although nasal septum surgery involves manipulating anatomical structures near the nerves supplying the anterior maxillary teeth, these sensory disturbances were typically temporary and resolved without long-term complications in most patients.

The incidence of postoperative sensory reduction in our study aligns with prior reports in the literature, which have cited numbness or paresthesia affecting the anterior maxillary teeth in 3%−30% of cases [13, 14]. In our cohort, approximately 10% of patients experienced genuine postoperative sensory disturbances, placing our findings at the lower end of this range.

Our study supports previous research suggesting that nerve injuries related to septal surgery are typically transient, with recovery observed within weeks to months. This is explained due to the ability of the nerves to regenerate or recover from minor trauma. For instance, the nasopalatine nerve, potentially injured during chiseling of the maxillary crest, showed recovery in the follow-up period, reflecting the nerve's capacity for healing. Another explanation is that the anterior superior alveolar nerves travel through a canal in the anteromedial maxillary wall, a site that is typically not affected by nasal septal manipulation.

Permanent sensory loss has been reported in the literature and attributed to more severe nerve damage due to cautery use near the nasopalatine foramen. The surgical techniques employed in this study—particularly the avoidance of extensive cauterization and careful manipulation of the septum near the incisive foramen—may explain the favorable outcomes seen in our patients.

We obtained dental radiographs for patients who exhibited reduced teeth sensation from the initial preoperative assessment (patients #4, #7, #9, #11, and #14). Although no pathological radiological findings were identified to explain the reduced sensation, several factors may account for this, such as a thick dentine layer or a slightly obliterated pulp chamber, which could explain the lack of response to cold stimuli.

Patients with a history of nasal fractures or previous nasal surgeries may be more prone to postoperative sensory changes due to preexisting anatomical alterations. In our study, 24% of patients had a history of nasal fractures, and 19% had undergone prior nasal surgery, yet these factors did not appear to be associated with postoperative sensory loss in the anterior teeth. Similarly, osteotomies were not found to correlate with sensory changes.

Several limitations of our study should be noted. First, the sample size was relatively small, limiting the generalizability of our findings. Although the low incidence of postoperative sensory changes in our study is encouraging, larger prospective studies are necessary to confirm these results and to better identify predisposing factors for postoperative numbness. Future research should also explore the impact of different surgical techniques, such as open rhinoplasty, and compare sensory outcomes across a broader range of surgical approaches.

Another limitation is the relatively short follow-up period, and the fact that patients with normal sensory examinations were not retested. Although no patients in our study experienced prolonged sensory disturbances, longer follow-up is needed to rule out late-onset complications or extended recovery timelines in rare cases.

In conclusion, nasal septal surgery is associated with a low incidence of transient sensory changes in the anterior maxillary teeth, with full recovery typically occurring within one to three months. These findings highlight the importance of careful surgical technique and preoperative counseling, while offering reassurance that permanent sensory loss is exceedingly rare. Further research with larger patient cohorts and longer follow-up is warranted to refine our understanding of this complication and optimize patient outcomes.

## Data Availability

The data supporting the findings of this study are available upon request from the corresponding author.
